# Recent update on crosslinked polyethylene in total hip arthroplasty

**DOI:** 10.1051/sicotj/2020013

**Published:** 2020-05-15

**Authors:** Jean Langlois, Moussa Hamadouche

**Affiliations:** 1 Centre Orthopédique Santy 24, avenue Paul Santy 69008 Lyon France; 2 Hôpital Privé Jean-Mermoz 55, avenue Jean-Mermoz 69008 Lyon France; 3 Département de Chirurgie Orthopédique, Université Paris-Descartes, Assistance Publique-Hôpitaux de Paris, Hôpital Cochin 27, rue du Faubourg Saint-Jacques 75014 Paris France

**Keywords:** Total hip arthroplasty, Highly crosslinked polyethylene, Wear, Oxidation, Fatigue resistance

## Abstract

More than two decades after their clinical introduction, crosslinked polyethylenes (XLPE) have been widely adopted. Though concerns were initially raised regarding oxidation and brittleness, on a large scale, the first generation of XLPE continues to be highly effective 15 years after the surgery, even in a young and active population. Remelted XLPE might display lower wear rates than annealed XLPE. Second generation XLPEs, not only including sequentially irradiated and annealed but also associated with antioxidants, demonstrate encouraging short- to mid-term results. Registry data support clinical trial reports. Even in less favorable settings (lipped liners, dual mobility cups, revision surgery, hip resurfacing) results are promising. However, failures (fractures) have already been described. Therefore, a high level of surveillance remains crucial.

## Introduction

Primary total hip arthroplasty (THA) has proven to be highly effective for end-stage hip osteoarthritis. The majority of implants are expected to last over 20 years. Driven by these results, there has been a decline in the average age of THA candidates. Younger patients represent a unique challenge, as they demand longer implant survivorship, while their physical activity creates increased cumulative stress on the implants. Besides, obesity is reaching epidemic proportions, with increasing demand for THA, and represents an additional challenge for the choice of bearing surface.

With the number of primary THA procedures expected to grow, and the cost-effectiveness being scrutinized, minimizing early complications, such as instability, while reducing wear and extending component survivorship, continues to draw heavy attention. Selection of the proper bearing material is therefore a key to optimize both short- and long-term results.

With the widespread adoption of crosslinked polyethylene (XLPE), particle osteolysis and aseptic loosening has become less common than in historical series, and other causes (dislocation, infection) have come to account for more than half of the revisions [1]. However, concerns with the long-term results of XLPE have been raised, due to risk of fatigue fracture, oxidation, and wear particles having a different bioreactivity profile that could result in more aggressive osteolysis, even in low wear rate settings. After two decades of utilization, it became essential to review some of the most relevant and recent clinical studies about XLPE.

## Long-term wear, osteolysis, and survivorship

Crosslinked polyethylene has demonstrated during the first decade by all means lower wear rates than conventional polyethylene (CPE) [2–4]. But publications are scarce regarding outcomes over 15 years of follow-up, when complications, such as wear or oxidation may occur.

At a mean follow-up of 16 years, Bryan et al. [5] reported results of 237 patients under 50 years of age (273 hips, 216 melted XLPE versus 57 CPE). Using a manual method, the mean linear CPE wear rate was 0.23 mm/year, while the XLPE group had no detectable wear. Forty-four patients (77%) in the CPE group had evidence of osteolysis compared to no osteolysis in the XLPE group. They were six revisions for wear in CPE group (10.5%) compared to none in the XLPE group (*P* < 0.001). Rames et al. [6] found similar results, evaluating 54 hips in a young population receiving a melted XLPE. At average 15 years, wear rate was 0.0185 mm/year. No liner fracture, no osteolysis, and no loosening were reported. The survivorship with all causes of revision as endpoint was 97.8%. Hopper et al. [7] compared the results of 230 hips randomized to receive either a 50 kGy melted XLPE or a CPE. The 15-year wear-related revision rate was lower in the XLPE group (0%) than in the CPE group (12%; *P* < 0.001). Among unrevised THAs, XLPE wear rate (0.03 mm/year) was lower than CPE wear rate (0.17 mm/year; *P* < 0.001). Osteolysis of any size was noted among 9% of the XLPE hips versus 46% of the CPE hips (*P* < 0.001). Similar results with melted XLPE outperforming CPE with at least 15 years of follow-up were reported by Moon et al. [8]. Since plain radiographs have a sensitivity close to 40% [9], osteolysis has been specifically assessed by Fukui et al. [10] with computed tomography and 3D multiplanar reconstruction images. Data from 105 primary THA using a melted XLPE liner (in an uncemented cup with two screws, against 26 mm zirconia femoral head) were extracted at a mean follow-up of 15.9 years. No obvious osteolysis in the acetabulum or femur in any patient was detected.

Gaudiani et al. [11] evaluated 57 consecutive hips with a mean follow-up of 14 years using annealed XLPE and found it to have excellent long-term behavior: a 0.032 mm/year wear rate, no observed osteolysis, no implant failure because of loosening on either the acetabular or femoral side. Conversely, Tsukamoto et al. [12] warned of possible excessive wear for an annealed XLPE between 10 and 15 years. This annealed XLPE demonstrated no advantage in the wear rate or in the incidence of osteolysis at 15 years, despite having superior wear resistance up to 10 years, as compared to CPE. No liner fracture occurred. The hypotheses were that, either the level of crosslinking was not sufficient enough to reduce wear beyond 10 years, or oxidation occurred, accelerating material failure and increasing wear rate. The degree of oxidation of the explants was unfortunately not analyzed. On a large scale, the first generation of XLPE continues to be highly effective 15 years after the surgery, even in a young and active population. Remelted XLPE might turn to display lower wear rates than annealed XLPE.

Gaudiani et al. [13] demonstrated encouraging results at mean follow-up of 6 years with the sequentially irradiated and annealed PE, with low wear rates (0.015 mm/year). Moreover, they were unable to detect a significant difference in wear rates with 32- or 36-mm, metal and ceramic heads. Radiographic analysis revealed no instance of osteolysis. Bonutti et al. [14] evaluated wear rate of the same sequentially irradiated and annealed PE with a mean follow-up of 10.3 years (118 patients, mean age of 63 years) and found it to be extremely low (0.014 mm/year), with no osteolysis. The 10-year survivorship was 98.3%. Deckard and Meneghini [15], using both ceramic and cobalt-chrome heads, from 32 to 40-mm diameters, found surprisingly higher wear rates of sequentially annealed PE (0.095 mm/year, with minimum 5-year follow-up), close to the 0.1 mm/year which is known as the osteolysis threshold.

Extended reviews have been published about XLPE including vitamin E or other antioxidants [16, 17], but to date, main data are collected from ex vivo experiments or clinical studies with less than 5-year follow-up. At this short term, vitamin E poly has not proved any clinically significant advantage in terms of patient function, wear or revision rate as compared to first generation of XLPE. Galea et al. [18] published data from a multicenter study assessing the midterm behavior of a vitamin E infused XLPE. Implant survivorship was 97.1% at 5 years. They found a very low wear rate (0.01 mm/year), with no evidence of osteolysis or aseptic loosening. Regression analysis showed that metal (versus ceramic) femoral head was predictive of increase wear, as opposed to cup position, femoral head size, or BMI. Lindalen et al. [19] measured at 6-year follow-up wear, with radiostereometric analysis in THAs articulating delta ceramic heads (32–36 mm) and vitamin infused XLPE. They found a mean 0.015 mm/year wear rate. As conclusion, Vitamin E XLPE has encouraging early results, but long-term follow-up will be required before definitive conclusions can be provided.

## Risk factors of wear

Numerous factors have been associated with greater wear using CPE. Whether these relationships hold true with XLPE is still a matter of debate. Regarding patient-related factors, Rames et al. [6] showed no statistically significant difference in terms of wear rates between a subgroup of highly active patients (UCLA score 8–10) compared to a lower demand group (UCLA score 1–7).

Lachiewicz et al. [20] evaluated at a mean follow-up of 11 years whether femoral head size had an influence on wear of melted XLPE. They reported about a cohort of 84 hips, using 26, 28, 32, 36 and 40 mm cobalt-chrome heads. As a result of surgical indications, the patients with 36-/40-mm head were significantly older (75 years) than those with smaller heads (58 years; *P* < 0.001). With the numbers available, the authors found no association between femoral head size and linear wear rate. The same authors then selected 107 hips (mean patient age 76 years) with larger heads (36–40 mm heads). At a mean follow-up of 8 years, they found no wear difference between 36 and 40-mm [21]. Likewise, Lindalen et al. [19] found no difference between 32 and 36-mm ceramic heads articulating in front of a vitamin E XLPE at 6-year follow-up.

In order to minimize the production of wear particles, different femoral head materials have been tested to articulate with XLPE. After 5-year follow-up, Jassim et al. [22] reported on 32 mm heads against melted XLPE and found no difference between oxidized zirconium and cobalt-chrome. Similarly, Sato et al. [23] showed no difference 6.3 years after the index surgery between cobalt-chrome, alumina and zirconia ceramic heads (22 or 26 mm diameters). With 12-year follow-up. Garvin et al. [24] found no difference in wear rates between cobalt-chrome, ceramic and oxinium with 28 mm heads. In conclusion, the 10-year cumulative revision rates reported in the Australian Registry (2016 Annual report) were similar for all three bearing materials on XLPE, at 3.2% for ceramicized metal, 4.4% for ceramic, and 4.3% for metal [25].

Cheung et al. [26] were the first to demonstrate, after mean follow-up of 13 years, a significant relationship between cup positioning (inclination and anteversion) and linear wear rates using melted XLPE. Except age and comorbidity, other patient demographics factors, such as gender, body weight, BMI, UCLA activity score, alcohol, or steroid intake, but also liner thickness or femoral offset, had no influence on wear rates in that study. Conversely, Moon et al. [8] failed to demonstrate any statistical correlation between wear and cup position. In conclusion, acetabular component orientation probably keeps affecting wear rate of XLPE liners. However, in contrast to CPE, it remains less evident that patients’ activity, head size, or material, play a clinically relevant role, based on the mid- to long-term studies currently available.

## Registry data

A recent report based on the Australian registry revealed a significantly lower cumulative rate of revision using XLPE (6.2%) when compared to CPE (11.7%) at average 16-year follow-up [27]. This difference prevailed within subgroup analyses, such as cup design, femoral head material or diameter. This discrepancy was further exaggerated when stratified for cohort of patients under 55 years of age (revision rate was 17.4% for CPE, versus 6.6% for XLPE). The authors concluded that the benefit of XLPE was evident both early and late, with a reduced rate of revisions due to dislocation (XLPE allowing a sound use of larger femoral heads) and to wear-related issues.

Similar differences were reported from the 2019 New Zealand Joint Registry [28]. They showed that using ceramic femoral heads, the revision rate per 100 component years for CPE was 0.77, in contrast to the XLPE rate of 0.54. For those cases using a metal femoral head the corresponding figures were 0.76 for the CPE and 0.56 for the XLPE.

Swedish registry data (from the 2013 report) demonstrated a lower overall risk of revision at 12 years for XLPE compared with CPE (1.9% and 4.3%, respectively) [29]. Regarding revision risk with at least 7.5 years of follow-up, data analyses from the Nordic Arthroplasty Register Association showed evidence that specific cup designs and fixation type (cemented or not) may affect the implant survivorship of the implant, as much as the PE formulation itself [30].

A recent study based on the UK National Joint Registry found that PE liners with a total radiation dose of ≥ 50 kGy demonstrate best survival at 14-year follow-up [31]. Moreover, highly irradiated liners (100 kGy or above) were not associated with an additional reduction in the risk of revision, when compared to moderately irradiated liners (50–100 kGy). Stabilization with vitamin E and heating above melting point performed best. To our knowledge, no long-term registry data are available regarding to XLPE including antioxidants.

## Less favorable settings

### Revision

Revision surgery exposes to third-body wear and less accurate component positioning. Lim et al. [32] evaluated 63 revision procedures using a melted XLPE. At 11-year follow-up, three hips had radiolucent lines around the cup, without evidence of loosening. On the femoral side, 5 hips had radiolucent lines in zones 1 and 7, but no subsidence. The mean linear wear rate was 0.029 mm/year. Five hips required re-revision, including 1 cup loosening, one recurrent dislocation, and 3 infections. None of the liners was revised due to polyethylene wear or mechanical failure. As confirmed by another publication [22], no difference was noted between metal and ceramic head when articulating with XLPE. Consequently, it appears that using an XLPE acetabular liner might be more important in reducing wear than the choice of femoral head bearing. It might represent an important key, when dealing in revisions with damaged tapers and the risk of ceramic head fracture.

### Elevated-rim and offset liners

Concerns about XLPE brittleness may raise with specific thin designs, such as elevated-rim or offset [33] liners. These constructs may be more vulnerable to rim cracking or fracture due to impingement, potentially leading to excessive wear and loosening. However, the evidence for this hypothesis remains yet inconclusive. Shin et al. [34] were among the first to compare standard versus elevated-rim melted XLPE liners. At 15-year follow-up, wear rates were low (<0.03 mm/year) and not significantly different between groups. Survivorships for all-cause reasons were excellent (>96%) and not significantly different. One case (1.3%) of osteolysis was confirmed in the standard group, whereas no osteolysis was observed in the elevated-rim group. Similarly, a recent analysis based on the Australian registry [35] found that XLPE lipped liners were not associated with an increased revision rate for aseptic loosening at a mean of 5 years. Revisions for breakage of the acetabular liner were extremely rare, with 11 for lipped liners (0.009%) and four for standard liners (0.006%). For Davis et al. [31] the use of asymmetrical (lipped) XLPE liners was actually associated with reduced risk of revision for any reason, for aseptic loosening (possibly the result of confounding variables, such as surgical approach, implant design and positioning), and for reasons other than aseptic loosening.

### Dual mobility

The concept of dual mobility is also mechanically demanding for the PE mobile insert, since it has to combine the theoretical wear resistance with sufficient elasticity to allow the initial passage of head through the restriction zone. These concerns were confirmed recently by biomechanical experiments [36]. They suggested that during the snap-fit head introduction, the impaction in force would overcome the ultimate strength of the XLPE. Therefore, nonreversible plastic deformation of the restriction zone and cracks would occur, leading to a loss of retentive power. The first finding was that femoral head snap-fit did not generate more or wider cracks in the retentive area of annealed or remelted XLPE than of CPE. Second important finding was that, as compared to CPE or annealed XLPE groups, for the remelted XLPE group, femoral head extraction force was significantly lower when cracks were present. It has to be determined if this difference is of clinical relevancy.

Epinette et al. [37] were the first to evaluate performance of a dual mobility acetabular system with XLPE (sequentially irradiated and annealed) with midterm follow-up. This multicenter prospective study included 321 young patients (mean age 48 years). There was no dislocation, nor any intra-prosthetic dissociation. Two acetabular shells were revised for neck-rim implant impingement without dislocation. Survivorship for all-cause was 97.5% at 5 years.

Short-term retrieval data from 33 XLPE (sequentially irradiated and annealed) dual mobility components (revised for non-mechanical failure) from D’Apuzzo et al. [38] suggested that although motion occurs at both bearing articulations, the inner bearing motion dominates. Second, the locking mechanism remained intact in the short term regardless of liner size. Lever out tests to dislocate the femoral head from the mobile PE (intraprosthetic dislocation) were performed on retrievals in order to challenge the locking mechanism. Results did not demonstrate any relationship between length of implantation (mean 6 months, range 0.06 to 26) and dislocation load. Taken altogether, these results suggest that a second generation of XLPE such as sequentially annealed or vitamin E-doped XLPE might probably constitute the most suitable material for dual mobility systems.

### Resurfacing

Encouraging midterm results have been reported with metal-on-XLPE hip resurfacing using “two-piece” acetabular components (a XLPE liner fixed in a titanium shell) [39, 40]. But these thick constructs can lead to undesirable acetabular bone removal, and expose to liner dissociation or thin XLPE fracture. For that purpose, Treacy et al. [41] recently reported preliminary data (88 hips, mean follow-up 1.6 years, 0.7–3.9) about a novel design of hip resurfacing arthroplasty using a direct-to-bone cementless mono-bloc XLPE component, in a cohort of 84 patients (73% of women, mean age 56 years) currently contraindicated for metal-on-metal resurfacing. No early failure occurred. The short-term functional results in this small cohort are similar with those of THA. Radiographs showed one head-neck junction radiolucency, but no osteolysis, component migration, or femoral neck thinning. However, wear and survival rates data with longer term follow-up will be needed.

## Crosslinked polyethylene failures

Crosslinking processes were developed to improve wear resistance, however balanced with brittleness and decreased fracture toughness. Several clinical reports have been published about fractures of XLPE liners ([42–44], non-exhaustive list). More recently Ast et al. [45] reviewed all the voluntary reports of one specific non-constrained, non-offset melted fractured liner to the US FDA, and confronted these findings to the current literature, to determine if any risk factors could be identified. The research was completed between 1999 and 2013. There have been 74 reports of fractured liners during this period, and all cases required revision surgery. The average time in situ was 27 months (range 1–96). No correlation was detected between the material thickness and the time in situ. Most cases (69%) reported small acetabular shells (≤54 mm) combined with large diameter heads (≥36 mm). Liners fractured were less than 7 mm thick at the weight bearing area (82% of the cases), and/or <4.7 mm thick at the rim (97% of the cases). It should be remembered that the difference in thickness between the weight-bearing area and the rim (the area that fractured in the majority of the cases) is more pronounced as the diameter of the femoral head increases. As an example, for a 52-mm shell, the difference is 1.6 mm for a 28-mm head, but raises up to 3.3 mm for a 36-mm head.

As a conclusion, fractures are likely to be multifactorial issues: the inherent mechanical characteristics of remelted HXLPE liners, liner/shell designs with stress concentrators [46, 47], trauma or dislocation, malposition of the shell, neck-liner impingement, and the use of thin polyethylene liners. These warnings should probably be extended to any PE formulation, even the more recent and resilient ones, since liner fractures have already been described [48, 49].

Also instructive in a different manner, is the failure report of a specific formulation of XLPE: a moderately cross-linked using two doses of 25 kGy, for a total irradiation dose of 50 kGy. No thermal treatment to quench free-radicals is applied. And the sterilization is based on gamma irradiation. This polyethylene liner has a specific design that is considered to be unique by the manufacturer, i.e. the “polar-locking” mechanism that is combined with antirotational tabs. Kahlenberg et al. [50] identified five cases (among 204 primary THA) of severe polyethylene wear (0.265 mm/year rate) and osteolysis which occurred within 5 years of the index surgery using this specific PE. Ceramic heads with 36-mm diameter were used in all the cases. All patients were men, with excellent early results and high activity scores (mean UCLA 7.4). Socket abduction was always less than 44°. Retrieved liners had grossly visible wear but no discoloration suggestive of oxidation. Similar reports (12 cases), with no identifiable factor, have been published recently [51], and the manufacturer revealed 22 reported cases of such failure between 2009 and 2019. Hypotheses for this usual early excessive wear are manufacturing characteristics of the polyethylene liner itself or the locking mechanism, or a combination of both. Further investigations are underway. This example proves that surgeon vigilance, along with registry data and scientific publication, continues to be invaluable.

## Conclusion

There is a long list of both outstanding and disastrous innovations in the field of hip replacement, especially with bearing materials (PTFE, Hylamer, Yttria-stabilized tetragonal zirconia heads, metal-on-metal) [52, 53]. The ideal bearing surface has yet to come [54]. The current evolution of PE might lead to one of them. Given optimistic estimates, the US might save one billion dollars over a 15-year duration with the use of XLPE [55], assuming this product would be responsible for a 40% reduced rate of all-cause revision.

But in the search for this breakthrough material, several statements should rule:

- Each PE is a product with a specific manufacturing process and should be evaluated individually. Pooling all the polys into generation boxes might be an irrelevant shortcut.
- Since THA standards have been brought to such a high level, any modification should come with solid arguments (i.e. long-term results, from non-designer teams) before widespread.
- Keeping a high level of post market surveillance is crucial, including prospective RCT, national registries data, but also retrieval analyses with standardized protocols.

There is no doubt that we should keep the innovation pipeline running. But a healthy skepticism should always prevail, by means of stepwise introduction of new technology [56].

## Conflict of interest

The authors declare that they have no conflict of interest.
